# Contextualizing condoms: a cross-sectional study mapping intersections of locations of sexual contact, partner type, and substance use as contexts for sexual risk behavior among MSM in Peru

**DOI:** 10.1186/s12879-019-4517-y

**Published:** 2019-11-11

**Authors:** R. Colby Passaro, Angelica Castañeda-Huaripata, Williams Gonzales-Saavedra, Susan Chavez-Gomez, Eddy R. Segura, Jordan E. Lake, Robinson Cabello, Jesse L. Clark

**Affiliations:** 1 Department of Emergency Medicine, Keck School of Medicine of the University of Southern California, Los Angeles, CA USA; 2 South American Program in HIV Prevention Research, David Geffen School of Medicine, University of California Los Angeles, Los Angeles, CA USA; 3 Asociación Civil Via Libre, Lima, Peru; 4 Escuela de Medicina, Universidad Peruana de Ciencias Aplicadas, Lima, Peru; 5 Department of Internal Medicine, Division of Infectious Diseases, McGovern Medical School at UTHealth, Houston, TX USA

**Keywords:** Condomless anal intercourse (CAI), Substance use, Men who have sex with men (MSM), Sex venue, Public health

## Abstract

**Background:**

Condomless anal intercourse (CAI) appears to be increasing among men who have sex with men (MSM) globally, and is reported to be as high as 70% in recent studies in Peru. To improve understanding of the evolving context of CAI among MSM in Peru, we studied associations between partner type, substance use, and condomless anal intercourse (CAI) in locations where MSM commonly report having sexual encounters.

**Methods:**

In a 2017 cross-sectional study of rectal STI screening and HIV prevention, a convenience sample of MSM recruited from community venues in Lima completed a survey of demographic characteristics and sexual risk behavior with their three most recent partners. Generalized estimating equations estimated correlations of CAI with location of last sexual contact, participant substance use prior to sex, and negotiation of condom use before or during sex. The network data integration application, Cytoscape, mapped intersections of partner type, sexual orientation, substance use, and CAI by four types of locations where sex occurred: 1) Home, 2) Hotel, 3) Sauna or Internet Cabin, and 4) Public Spaces.

**Results:**

Of 447 MSM (median age 27 years), 76.9% reported CAI with ≥1 of their last three partners. Participants reported sex with casual partners most commonly in homes (64.6%) and hotels (60.4%), and with anonymous partners most often in saunas/Internet cabins (57.5%) and public spaces (52.6%). CAI was less commonly reported in hotels (aPR, 95% CI: 0.85, 0.75–0.97) compared to homes. Participants who used marijuana before sex at home were more likely to report CAI than MSM who did not use marijuana (1.36, 1.01–1.92). Partner alcohol use before sex was associated with CAI in saunas/Internet cabins (3.17, 1.45–6.91) and public spaces (2.65, 1.41–4.98). In the sexual network maps, almost all MSM who used drugs prior to their sexual encounters used drugs with more than one of their last three partners.

**Conclusions:**

CAI was common and associated with different risk factors, like partner type and substance use, based on location where sex occurred. Novel combination HIV, STI, and substance use prevention interventions must consider how the social environments of MSM influence condom use and other sexual risk behaviors.

**Trial registration:**

ClinicalTrials.gov Identifier NCT03010020, January 4, 2017.

## Background

The prevalence of HIV in men who have sex with men (MSM) in Peru is estimated at 15.2%, an alarming 50-fold higher than in the general population [1]. While HIV prevention programs have followed local epidemiology in targeting MSM, the prevalence of HIV among Peruvian MSM remained stable between 2002 (13.9%) and 2016 (15.2%) [1, 2]. Moreover, in studies as recent as 2016, 69–70% of MSM in Peru reported condomless anal intercourse (CAI) with one or more partners in the last 3 months [3, 4]. While a few studies worldwide have explored how the location where a sexual contact occurs influences sexual risk-taking behavior among MSM, the potential influence of different social environments on condom use by MSM in Peru has not yet been studied [5]. A better understanding of how circumstantial factors like partner type and substance use vary depending on the location of the sexual encounter is needed to inform combined HIV and sexually transmitted infection (STI) prevention interventions that address the evolving contexts of CAI in same-sex male partnerships in Peru.

Previous studies of sexual venues and other locales frequented by MSM in developed countries suggest socioenvironmental factors are associated with sexual risk-taking, highlighting the particularly high-risk intercourse at commercial sex venues [5–9]. Factors associated with attending sex venues include reporting a high number of male sexual partners, sex while on methamphetamine and/or marijuana, group sex, and CAI with a partner whose HIV status was unknown [5, 10, 11]. While few studies out of lower-middle income countries have addressed correlations between CAI and location where sex occurred, a 2017 study in Tijuana, Mexico found higher levels of CAI were associated with more frequent contacts with sexual partners at public venues in the last 2 months [12]. Importantly, these findings have been successfully translated into community-level behavioral HIV prevention interventions to reduce CAI among high-risk MSM in some settings [13–15]. A 2018 study showed that venue-based HIV and syphilis testing was feasible in Lima, and that testing at sex work venues yielded a 47% syphilis prevalence compared to 28% in other venues [16].

Potential consequences of condomless sex for MSM in Peru include increases in the incidence of STIs. Prevalence of bacterial STIs among MSM in Lima are extremely high, with recent estimates ranging from 7.4–13.3% for syphilis [17, 18] and 29.5–32.8% for gonorrhea (GC) and/or chlamydia (CT) at any anatomic site [19, 20]. Moreover, prevalence of Herpes Simplex Virus-2 (HSV-2) in this vulnerable population has been reported between 35.7 and 40.8% [17, 21]. Recently, innovative interventions like expedited partner therapy (EPT) have shown the potential to reduce community prevalence of STIs by targeting the highest-risk people in MSM sexual networks [22]. In a similar way, our current analysis aims to inform STI prevention and treatment intervention implementation by identifying the intersection of social and behavioral factors that create the highest-risk environments for condomless sex among MSM.

A detailed understanding of factors associated with patterns of CAI at different locations where MSM in Peru commonly have sex is critical for targeting HIV and STI prevention messaging and resource distribution to the areas of greatest need. Our study describes CAI prevalence and explores individual- and partner-level correlates at four commonly reported types of locations where sex occurs in a sample of Peruvian MSM. Our approach considers each site as a potential context for sexual risk behaviors like CAI, as well as for protective behaviors like discussions of HIV serostatus and condom use. We highlight variations and similarities between alcohol and drug use at different types of locations where sex occurs through visual depictions of the sample sexual network.

## Methods

### Participants and recruitment

Participants were selected from community venues by peer recruiters at Via Libre, a community-based organization in Lima that provides integrated sexual health services, as part of the screening process for a 2017 study of rectal STI screening and HIV prevention among MSM and transgender women (TW) in Peru. Enrollment in the screening protocol was limited to individuals who: 1) were at least 18 years old, 2) were assigned male sex at birth, 3) had not previously tested positive for HIV infection, and 4) reported at least one episode of condomless receptive anal intercourse (cRAI) with an HIV-infected or unknown serostatus partner in the previous 6 months.

### Study measures and procedures

Participants completed a computer-assisted self-interview (CASI) survey addressing participants’ demographic characteristics and sexual risk behaviors. Survey questions asked participants to describe their sexual orientation (heterosexual, bisexual, homosexual) and role (*activo* [insertive], *pasivo* [receptive], *moderno* [versatile], or other), as well as the physical location of their three most recent sexual encounters. Multiple-choice options for the type of location where sex occurred included, “Your Home,” “Your Partner’s Home,” “Sauna,” “Hotel,” “Internet Cabin,” “Public Space,” and an “Other” write-in option. For analysis purposes, locations where sex occurred were defined as one of four types: 1) Home: “Your Home” or “Your Partner’s Home” (e.g., private non-commercial venues), 2) Hotel (e.g., private commercial venues), 3) Sauna or Internet Cabin (e.g., semi-public commercial venues),^1^ and 4) Public Spaces (e.g., public non-commercial venues). All “Other” responses were reviewed with local study staff and re-coded appropriately. The most commonly reported “Other” responses were: “A Friend’s House” (*n* = 5), recoded as Home; and “A Beauty Salon” (*n* = 5), “Club” (*n* = 4), and “Meeting Place” (*n* = 4), all of which were recoded as Public Spaces.

These location types were selected because they were the most commonly reported locations where sex occurred among MSM in our study. Moreover, they represent a broad spectrum of features known to influence sexual risk behavior, including public to private and non-commercial to commercial spaces [5]. Previous studies of MSM in other international settings also suggest that the key factors of partner type and substance use explored in our study vary according to the locations where MSM perform sexual acts [6].

Partner characteristics and partner-specific sexual acts with each of the three most recent contacts were assessed by participant report. Questions elicited partner type (stable, casual, anonymous, transactional), type of intercourse (anal, vaginal, oral), sexual position during intercourse (insertive, receptive, both), condom use during each act, and event-specific alcohol and drug use by both participants and partners. Alcohol use questions distinguished between no alcohol consumption, some alcohol consumption, and being intoxicated. Drug use questions asked about use of marijuana, cocaine, heroin, methamphetamine, and/or poppers (amyl nitrates).

Study physicians performed a medical history and physical exam to assess for signs or symptoms of STI, and collected blood and rectal swabs for gonorrhea, chlamydia, syphilis, and HIV testing. Participants received on-site treatment for symptomatic rectal STIs, if noted during the exam, according to 2010 CDC guidelines [23]. Treatment for syphilis was consistent with stage of infection, as determined by the study physician following review of the participant’s prior history of syphilis, previous rapid plasma reagin (RPR) titer(s), and antibiotic treatment history. Participants diagnosed with HIV and/or STI were counseled on the importance of partner notification and provided information on local HIV/STI testing and treatment resources. All participants were compensated 15 *Nuevos soles* (approximately US $5.00) for transportation and provided with five condoms and sachets of lubricant at each visit.

### Consent/permissions

The Institutional Review Boards of the University of California, Los Angeles and *Asociación Civil Via Libre* reviewed and approved all study procedures prior to the initiation of study activities. Written informed consent was obtained from all participants prior to participation.

### Data analysis

Due to the likely influence of commercial and other unique motivations in transactional CAI encounters (*n* = 103), and due to previously observed differences in the social contexts of sexual risk behavior between MSM and TW, analyses were limited to contacts with non-transactional sexual partners by cis-gender male participants.

We constructed five multivariable regression models for the following sexual risk behaviors: 1) Participant alcohol use before or during sex; 2) Participant drug use before or during sex; 3) Knowledge of partner serostatus; 4) Conversations about condom use before/during sex; and 5) Receptive and/or insertive CAI. We constructed four additional models for the primary outcome of CAI stratified according to the location where sex occurred to explore how associations of sexual risk behaviors with CAI varied by venue. Variables were selected for inclusion in multivariable regression models based on conceptual reasoning [24–28]. All models were adjusted for participant age, education, and sexual orientation, partner type, alcohol and drug use by participants and partners, knowledge of partner HIV serostatus, and condom conversations before/during sex. Models were not adjusted for the use of heroin, methamphetamine, or poppers because their use was reported in less than 2 % of sexual encounters.

To measure the association between independent variables and dichotomous outcomes, we computed prevalence ratios with Poisson regression analyses with robust estimation of standard errors [29]. This application provides a more easily interpretable and better alternative to logistic regression, which produces an odds ratio and can consequently overestimate the prevalence ratio in cross-sectional studies. All multivariable analyses were conducted at the partner level, for which each of the three most recent partners (or, for repeat partners, the last sexual encounter with that partner) was the unit of analysis. Models were constructed under the generalized estimating equation extension with an exchangeable working correlation structure to account for correlation between the last three partners reported by the same participant [30].

A figure was designed using Cytoscape (Cytoscape Consortium, New York, NY) in order to provide a visual structure to the complex interactions between substance use, CAI, and the location where sex occurred, which cannot be clearly described by text or depicted in tables. Examining network connections among locations in this way yields a rarely considered additional level of information, identifying locations with high frequency of CAI and other risk behaviors that are highly connected [31].

**Fig. 1:**
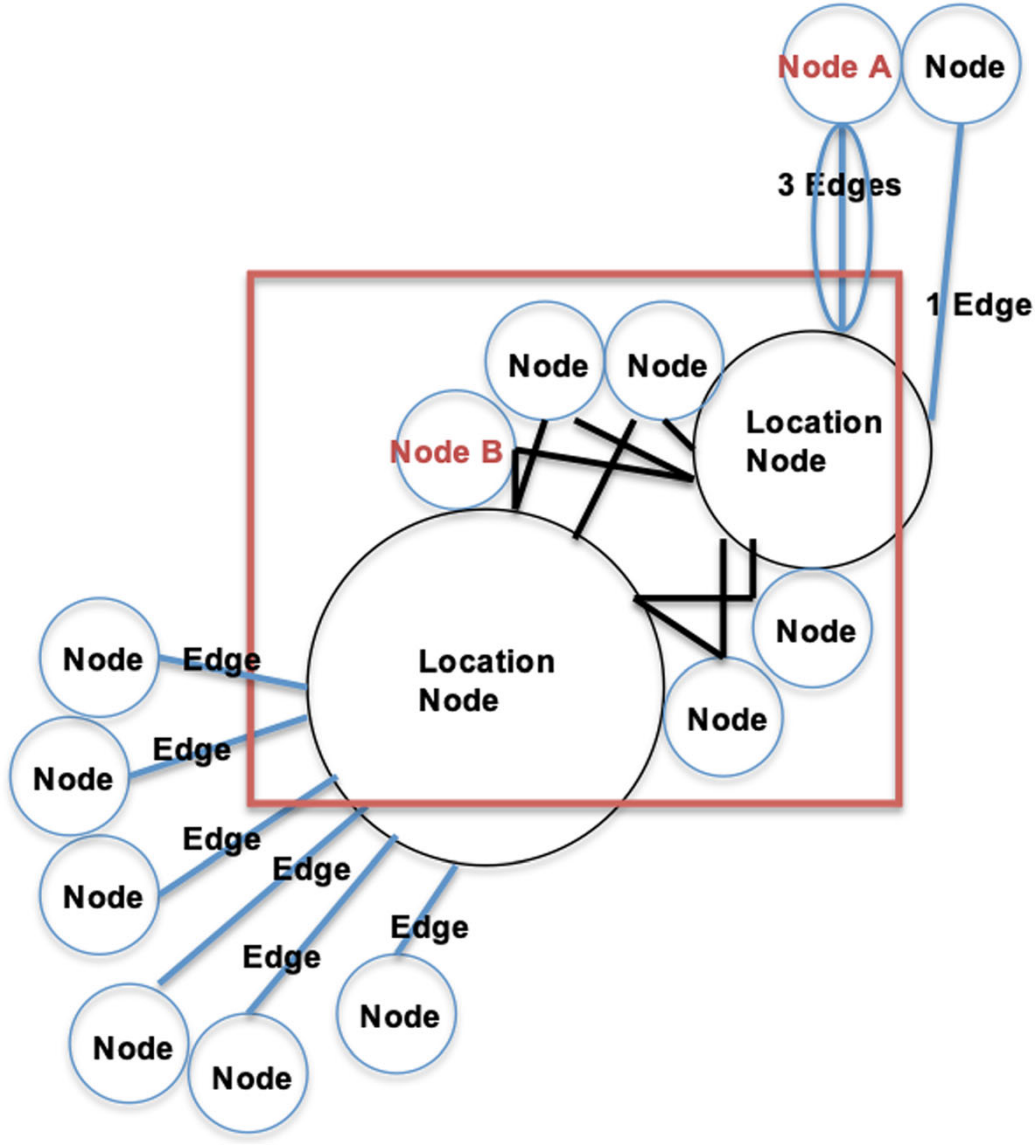
Labeled Cytoscape Example

Figure 1 provides a simplified, labeled example of the novel Cytoscape approach. In this example, each participant is represented as one node. Edges (up to three per participant) are undirected lines between participants and locations where sex occurred, with each edge representing one sexual encounter. Participants who had anal intercourse at more than one site appear in the center of the diagram (e.g., Node B has two edges [sexual encounters], one connecting him to each location), while participants endorsing anal intercourse at only one site are positioned outside of the circle (e.g., Node A has three edges [sexual encounters], each one connecting him to only one location). Each location is also represented as one node. The relative size of location nodes reflects the number of edges (sexual encounters) at that site (9 at the smaller node; 11 at the larger node, in this example).

All analyses were conducted using Stata 12.0 (StataCorp, College Town, TX). Complete case analysis was performed for variables with missing data; less than 5% of data were missing for any single variable.

## Results

### Sample characteristics

We evaluated a total of 447 MSM (median age 27 years) between July and December, 2017 (Table 1). The most frequently reported sexual orientation among participants was homosexual (82.2%, 361/447) and the most commonly reported sexual role was *moderno* (versatile; 51.1%, 226/447). About half of all participants met AUDIT criteria for an alcohol use disorder (AUD; 57.3%, 256/477) and used alcohol prior to sex with at least one partner (47.3%, 211/477). Participant drug use before sex with at least one partner (11.7%, 52/477) was less common than alcohol use prior to sex. More than two-thirds of the sample (76.9%, 343/477) reported CAI with one or more of their last three sex partners.

**Table 1:** Characteristics of MSM Participants in Lima, Peru, 2017; *N* = 447

Characteristic	Median (IQR^a^) or N (%)
Age (*n* = 446)	27 (22, 34)
Education (*n* = 446)	
< Secondary	29 (6.5)
Completed Secondary	154 (34.5)
> Secondary	263 (59.0)
Monthly income less than Lima average (*n* = 368)	
Yes	234 (63.6)
No	134 (36.4)
Sexual orientation (*n* = 439)	
Hetero/Bisexual	78 (17.8)
Homosexual	361 (82.2)
Sexual role (*n* = 442)	
Activo	9 (2.0)
Pasivo	207 (46.8)
Moderno	226 (51.1)
Type of location where sex occurred with ≥1 of last 3 partners (*n* = 447)	
Home	335 (79.4)
Hotel	247 (55.3)
Sauna/Internet cabin	56 (12.5)
Public space	35 (7.8)
Meet criteria for an alcohol use disorder (*n* = 447)	
Yes	256 (57.3)
No	191 (42.7)
Alcohol use before sex with ≥1 of last 3 partners (*n* = 446)	
Yes	211 (47.3)
No	235 (52.7)
Drug use before sex with ≥1 of last 3 partners (*n* = 446)	
Yes	52 (11.7)
No	394 (88.3)
CAI with ≥1 of last 3 partners (*n* = 446)	
Yes	343 (76.9)
No	103 (23.1)

^a^ *IQR* Interquartile range

**Fig. 2:**
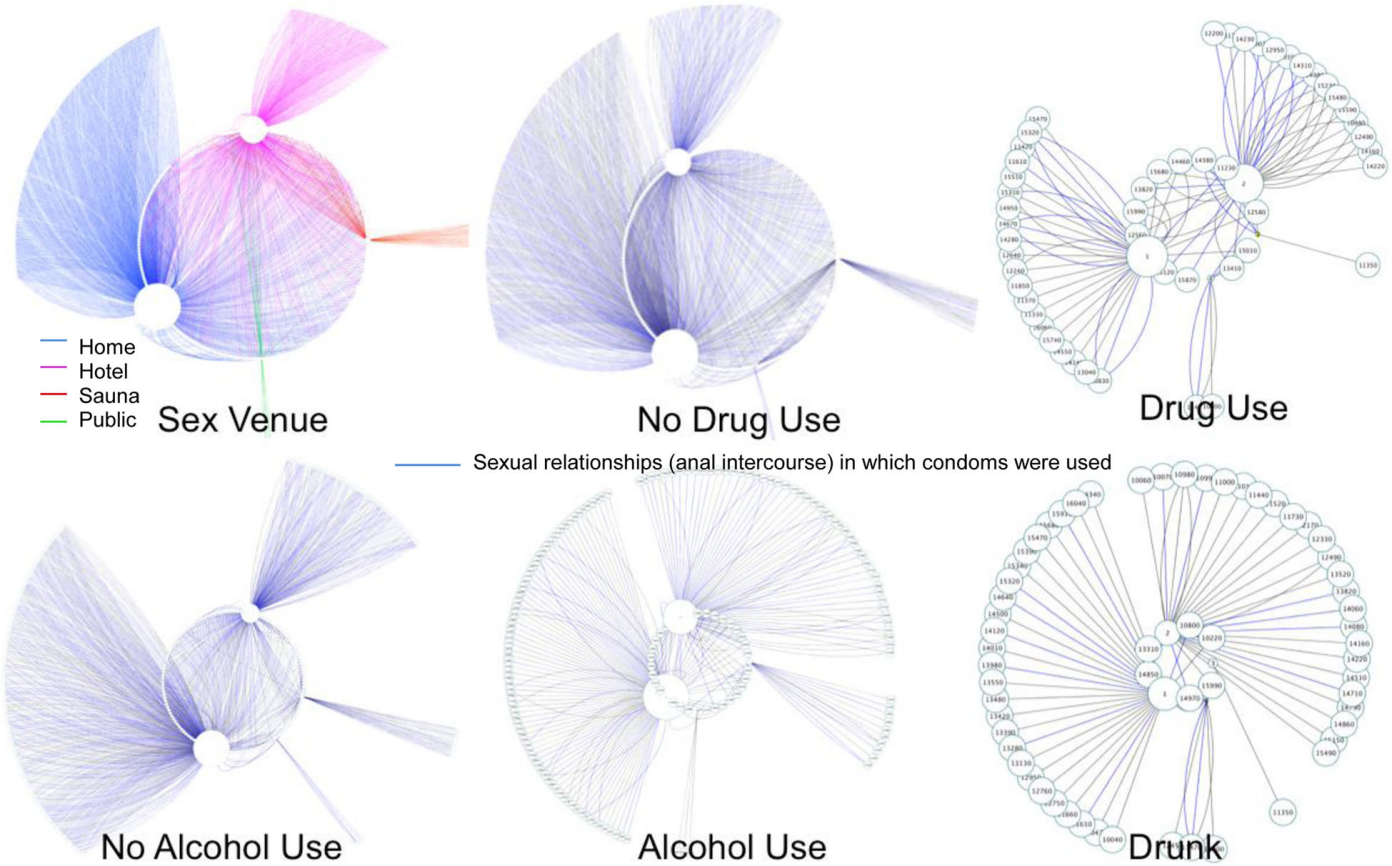
Patterns of Locations Where Sex Occurred Overall and by Participant Sexual Risk Behaviors; *N* = 1341. LEGEND: Venues are represented in each figure in the same order: home, hotel, sauna, and public.

Locations where sex occurred with one or more of the last three partners were: A home (79.4%, 335/447); A hotel (55.3%, 247/447); A sauna or Internet cabin (12.5%, 56/447); and A public space (7.8%, 35/477). Participants reported sex with casual partners most commonly in homes (64.6%, 448/722) and hotels (60.4%, 236/424), and with anonymous partners most often in saunas/Internet cabins (57.5%, 46/85) and public spaces (52.6%, 20/42). With stable partners, 65.5% (76/116) of sexual encounters were reported in homes, 32.8% (38/116) in hotels, and only 0.9% (1/116) each in saunas/Internet cabins and public spaces. Figure 2 shows patterns of locations where sex occurred overall.

### Sexual risk behaviors by location where sex occurred

In our sample of MSM, substance use by participants and partners prior to intercourse was most commonly reported in association with sexual encounters outside of the home, particularly in public spaces and hotels (Table 2). For example, 17.1% (7/42) of participants and 14.6% (6/42) of partners described themselves as “intoxicated” during their sexual encounters in public spaces versus 4.6% (32/722) of participants and 4.2% (29/722) of partners in home-based encounters (Table 2). Moreover, 7.9% (32/424) of participants and 8.9% (36/424) of partners smoked marijuana prior to sexual encounters in hotels, compared to 3.7% (26/722) of participants and 5.5% (38/722) of partners during home-based sexual contacts.

**Table 2:** Sexual Behaviors of MSM and their Last Three Non-Transactional Partners, Stratified by the Location Where Sex Occurred; *N* = 1341

Behavior	Total (*n* = 1273)	Home (*n* = 722)	Hotel (*n* = 424)	Sauna/Internet Cabin (*n* = 85)	Public Space (*n* = 42)
PARTICIPANT BEHAVIORS					
Substance use before/during sex (*n* = 1279)					
Alcohol – Intoxicated	78 (6.1)	32 (4.6)	33 (8.1)	1 (1.2)	7 (17.1)
Alcohol – Not intoxicated	260 (20.3)	127 (18.2)	97 (23.8)	18 (21.7)	6 (14.6)
No Alcohol	941 (73.6)	537 (77.2)	277 (68.1)	64 (77.1)	28 (68.3)
Marijuana	67 (5.5)	26 (3.7)	32 (7.9)	3 (3.6)	6 (14.6)
Cocaine	47 (3.9)	21 (3.0)	16 (3.9)	4 (4.8)	6 (14.6)
Knowledge of partner serostatus (*n* = 1273)	80 (6.3)	44 (6.1)	26 (6.1)	5 (5.9)	5 (11.9)
Condom conversation before/during sex (*n* = 1273)	154 (12.1)	92 (12.7)	48 (11.3)	8 (9.4)	6 (14.3)
CAI^a^ (*n* = 1226)	653 (53.3)	388 (55.8)	195 (48.0)	51 (61.5)	19 (46.3)
PARTNER CHARACTERISTICS/BEHAVIORS					
Partner type (*n* = 1203)					
Stable	116 (9.6)	76 (10.9)	38 (9.7)	1 (1.3)	1 (2.6)
Casual	734 (61.0)	448 (64.6)	236 (60.4)	33 (41.2)	7 (44.7)
Anonymous	353 (29.3)	170 (24.5)	117 (29.9)	46 (57.5)	20 (52.6)
Substance use before/during sex (*n* = 1227)					
Alcohol – Intoxicated	58 (4.7)	29 (4.2)	20 (4.9)	3 (3.6)	6 (14.6)
Alcohol – Not intoxicated	263 (21.4)	133 (19.1)	110 (27.0)	14 (16.9)	6 (14.6)
No Alcohol	906 (73.8)	534 (76.7)	277 (68.1)	66 (79.5)	29 (70.7)
Marijuana	88 (7.2)	38 (5.5)	36 (8.9)	5 (6.0)	9 (22.0)
Cocaine	50 (4.1)	20 (2.9)	19 (4.7)	4 (4.8)	7 (17.1)

^a^*CAI* Condomless anal intercourse

As seen in Fig. 2, the relative number of sexual encounters occurring outside of the home increases from left to right, highlighting the preponderance of substance use prior to intercourse in non-home sex venues. Moreover, the relative frequency of blue edges (representing anal intercourse protected by a condom) decreases from left to right, underlining the higher frequency of CAI in the context of alcohol or drug use. Finally, almost all of the target nodes in the “Drug Use” figure are connected to their respective source nodes by multiple edges, suggesting that MSM who used drugs prior to their sexual encounters tended to use drugs with *more than one* of their last three partners. This observation is in contrast to participants who endorsed being intoxicated prior to sex, who typically reported this behavior with *only one* of their last three partners.

### Multivariable analyses of sexual risk behaviors

Participant and partner substance use were highly associated. Participant alcohol use before/during sex was associated with partner alcohol use before/during sex, whether the partner was intoxicated (aPR, 95%CI: 18.8, 13.30–26.60) or not (17.4, 12.45–24.21; Table 3). Participant use of any drug before sex was also associated with partner marijuana use before sex (7.64, 3.53–16.54).

**Table 3:** Crude and Adjusted Poisson Regression Models for Sexual Behaviors with Non-Transactional Partners of MSM; *N* = 1341

	Participant alcohol use before/during sex (*n* = 338)		Participant drug use before/during sex^b^ (*n* = 101)		Knowledge of partner serostatus (*n* = 80)		Condom conversation before/during sex (*n* = 154)		Receptive/insertive condomless anal intercourse (*n* = 674)	
Characteristic	PR	aPR	PR	aPR	PR	aPR	PR	aPR	PR	aPR
Age	**1.02**	1.00	0.99	–	1.00	–	0.98	0.99	1.00	–
Education (<Secondary is the reference category)										
Secondary	0.81	0.92	1.65	–	0.61	0.73	0.86	–	1.01	–
> Secondary	**0.69**	1.01	0.57	–	0.59	0.86	1.07	–	1.06	–
Participant sexual orientation (Homosexual is the reference category)										
Hetero/Bisexual	0.82	–	1.49	–	1.15	–	1.19	–	**0.78**	0.82
Partner type (Casual is the reference category)										
Anonymous	**0.73**	0.98	0.74	–	1.32	–	0.99	0.99	**0.88**	0.89
Stable	0.93	1.08	0.85	–	1.12	–	1.37	1.33	0.92	0.93
Location where sex occurred (Home is the reference category)										
Hotel	**1.26**	1.07	1.13	–	1.12	1.10	0.89	–	**0.86**	**0.85**
Sauna/Internet cabin	0.91	1.17	0.98	–	0.91	1.32	0.69	–	1.06	1.10
Public space	1.24	1.05	1.42	–	1.89	1.13	1.00	–	0.90	0.88
Participant alcohol use before/during sex^a^										
Yes – Not intoxicated	–	–	**1.81**	1.64	0.69	–	1.01	–	**1.15**	0.99
Yes – Intoxicated	–	–	**3.35**	1.85	1.13	–	0.93	–	**1.33**	1.21
Partner alcohol use before/during sex										
Yes – Not intoxicated	**18.3**	**17.4**	**1.79**	0.88	0.87	–	0.96	–	**1.23**	**1.21**
Yes – Intoxicated	**19.8**	**18.8**	**3.03**	1.12	1.02	–	1.00	–	1.21	1.01
Participant drug use before sex										
Marijuana	**2.12**	1.13	–	–	**2.85**	1.74	1.27	–	1.21	1.24
Cocaine	**2.84**	1.19	–	–	**3.98**	**2.70**	1.89	1.42	1.10	–
Partner drug use before sex										
Marijuana	**2.13**	1.01	**9.83**	**7.64**	1.74	0.99	0.85	–	1.13	–
Cocaine	**2.71**	0.95	**6.89**	2.05	**2.95**	0.90	1.62	0.84	1.04	–
Knowledge of partner serostatus										
Yes	–	–	–	–	–	–	**4.77**	**4.68**	1.10	–
Condom conversation before/during sex										
Yes	–	–	–	–	**6.26**	**6.32**	–	–	**1.22**	**1.20**

**Bold text** = *p*-value < 0.05. Adjusted models include all variables with crude *p*-value < 0.20

^a^No is the reference value for all variables except Location where sex occurred, Education, Partner Type, and Participant sexual orientation

^b^Drugs include: marijuana, cocaine, poppers, methamphetamine, and heroin

Knowledge of partner serostatus was associated with condom conversations before/during sex (6.32, 3.77–10.59) and vice versa (4.68, 3.15–6.95), suggesting that these protective behaviors often clustered together. CAI was associated with alcohol use by the partner (1.21, 1.01–1.46) prior to sex, and was less commonly reported in hotels (0.85, 0.75–0.97) compared to homes.

### Multivariable analysis of CAI stratified by sex venue

Participant and partner alcohol use were associated with CAI in every type of location where sex occurred except the home. For example, in hotels, participants who reported being intoxicated before/during sex were more likely to endorse CAI than MSM denying alcohol use prior to sex (1.57, 1.02–2.43; Table 4). Meanwhile, partner alcohol use before/during sex was associated with CAI in saunas and Internet cabins (3.17, 1.45–6.91) and in public spaces (2.65, 1.41–4.98). In addition, participants who used marijuana before sex at home were more likely to report CAI than MSM who did not smoke marijuana (1.36, 1.01–1.82).

**Table 4:** Poisson Regression Models for CAI with Non-Transactional Partners of MSM, Stratified by the Location Where Sex Occurred; *N* = 1341

	Home (*n* = 722)			Hotel (*n* = 424)			Sauna/Internet Cabin (*n* = 85)			Public Space (*n* = 42)		
Characteristic	PR	aPR	95% CI	PR	aPR	95% CI	PR	aPR	95% CI	PR	aPR	95% CI
Age	1.00	–	–	1.00	–	–	0.98	0.98	0.96–1.01	1.01	–	–
Education (<Secondary is the reference category)												
Secondary	0.97	–	–	1.19	–	–	**0.56**	**0.49**	**0.33–0.72**	–	–	–
> Secondary	1.06	–	–	1.24	–	–	**0.60**	**0.58**	**0.42–0.81**	–	–	–
Participant sexual orientation (Homosexual is the reference category)												
Hetero/bisexual	**0.76**	0.78	0.61–1.01	0.86	–	–	0.85	–	–	0.51	0.89	0.21–3.84
Partner type (Stable is the reference category)												
Casual	0.89	0.90	0.76–1.07	1.16	–	–	0.81	–	–	0.54	–	–
Anonymous	0.94	0.95	0.76–1.20	1.05	–	–	–	–	–	–	–	–
Participant alcohol use before/during sex^a^												
Yes – Not intoxicated	**1.19**	1.04	0.81–1.32	1.24	1.25	0.83–1.89	0.84	0.52	0.25–1.09	1.52	–	–
Intoxicated	**1.35**	1.14	0.81–1.62	**1.51**	**1.57**	**1.02–2.43**	**1.60**	0.45	0.20–1.02	1.46	–	–
Partner alcohol use before/during sex												
Yes – Not intoxicated	**1.26**	1.17	0.91–1.52	1.24	0.97	0.64–1.46	0.96	**1.70**	**1.09–2.67**	**2.66**	**2.65**	**1.41–4.98**
Intoxicated	**1.29**	1.14	0.79–1.66	1.30	0.84	0.48–1.47	**1.67**	**3.97**	**1.83–8.64**	1.45	0.68	0.14–3.30
Participant drug use before sex												
Marijuana	1.32	**1.36**	**1.01–1.82**	1.26	–	–	–	–	–	1.24	–	–
Cocaine	0.87	–	–	**1.68**	1.34	0.82–2.17	0.87	–	–	1.12	–	–
Partner drug use before sex												
Marijuana	1.04	–	–	1.10	–	–	1.09	–	–	1.64	1.31	0.72–2.39
Cocaine	0.83	–	–	**1.48**	1.10	0.65–1.84	0.92	–	–	1.33	–	–
Knowledge of partner serostatus												
Yes	1.13	–	–	1.11	–	–	0.65	–	–	**1.70**	**3.56**	**0.62–20.59**
Condom conversation before/during sex												
Yes	1.15	1.13	0.93–1.37	1.28	**1.32**	**1.02–1.71**	1.22	–	–	1.40	1.08	0.28–4.24

*CAI* condomless anal intercourse

**Bold text** = *p*-value < 0.05. Adjusted models include all variables with crude *p*-value < 0.20

^a^No is the reference value for all variables except Education, Participant sexual orientation, and Partner type

## Discussion

Among our sample of MSM in Lima, CAI with one or more of the last three non-transactional partners was common and associated with factors that varied according to the location where sexual contact occurred. Taken together, these findings suggest the need for a more nuanced approach to understanding the complex interaction of location type, subject and partnership characteristics, substance use, and sexual behavior in defining event-specific HIV/STI transmission risk. For example, while substance use was associated with CAI across all locations where sex occurred, the type of substance used and the “user” varied by location. Moreover, while CAI was less common in some settings compared to others, participant and partner substance use were highly associated with each other regardless of the location where sex occurred, and were variably associated with CAI in different location types. In this way, location type may be as much a part of the sexual network as the MSM connecting there, and is likely a critical part of the complex hierarchy of decisions surrounding sexual risk behavior, including cultural and network norms and individual risk psychology [32]. These findings support the differentiation of private versus public venues as contexts of sexual risk behavior, reinforce the powerful role of substance use in CAI among MSM, and highlight the potential utility of combination STI/HIV/substance use prevention techniques that can be adapted to the specific physical locations of male-male sexual interactions.

Reflecting the diversity of location-specific patterns of substance use and sexual behavior in our sample, alcohol use was associated with CAI in every type of location where sex occurred except the home, where participant marijuana use was associated with CAI. While a previous study in the U.S. identified an association between marijuana use prior to sex in commercial and public sex venues, our results suggest that the familiarity of the home environment may encourage casual drug use as well as sexual risk behavior. As a result, counseling about the risks of sex in the context of marijuana cannot be limited to MSM who attend high-risk public venues, and need to address the potential for HIV and STI transmission risk even in what are perceived as “safe” spaces [5]. These findings also emphasize the importance of the association between alcohol use and CAI found in other studies of MSM in Peru, showing that this association cuts across multiple social contexts [24, 33]. Our study is the first to identify an association between marijuana use and CAI among MSM in Peru. While there are few studies addressing the use of marijuana and sexual risk behavior among MSM worldwide, marijuana has been shown to alter judgment and impair motor coordination, and may increase the likelihood of engaging in sexual behaviors that facilitate STI and HIV transmission [34]. Future qualitative studies are needed to better understand how social environments influence the alcohol and drug use choices of MSM, how these decisions affect actual and perceived event-level risk for HIV/STI transmission, and how substance use management can be integrated with HIV/STI prevention.

Another key finding in our study was that most MSM who endorsed drug use prior to intercourse did so with two or more of their last three partners, suggesting a concentrated pattern of drug use among a sub-group of MSM. Combined with the finding that participant and partner drug use were highly correlated, our results suggest that a concerted drug use treatment intervention targeted to the highest-risk MSM could have a disproportionate, positive effect on community-level substance use. While illicit drug use among MSM in this and previous studies in Peru is only around 10%, drug use in this population has been associated with risky sexual behaviors, including high numbers of sexual partners and CAI [25]. Interventions addressing impulsive delay discounting may effectively address such risk clustering as this form of economic behavioral decision-making may have a role in both sexual risk taking and substance use [35]. A 2013 intervention in South Africa aimed towards men and their drinking environment was able to foster lasting HIV and alcohol behavior change, suggesting that interventions that address both the social environment and community norms may be capable of producing durable substance use and sexual risk behavior changes [36]. As little is known about drug use among MSM in Peru, further research is needed to characterize patterns of substance use in specific social, environmental, and partnership contexts, and to identify the different effects of specific drugs on both sexual behavior and biological vulnerability to HIV/STI acquisition and transmission in this population [37–39].

As predicted, distinct patterns of association between partner type and location of sexual contact were observed in our sample: most partners in homes and hotels were casual, and most partners in saunas/Internet cabins and public spaces were anonymous. These trends reflect the results of studies in developed countries identifying commercial sex venues as high-risk environments characterized by anonymous encounters [5]. However, our results also identify a novel area for intervention because MSM in our sample were less likely to report CAI with partners in hotels than in homes. While this finding may in part be explained by MSM inviting casual partners with whom they are more acquainted into their homes, it also highlights the danger of the perceived safety of repeat casual partners. In addition to further highlighting the potential risk of infectious disease transmission in environments perceived as “safe,” this finding also provides a more detailed understanding of the role of the site of sexual contact than previous studies of CAI and place in Latin America, which limited their analyses to commercial and/or public sex venues [12, 40]. Successful combination HIV and STI prevention interventions therefore cannot focus only on traditionally high-risk sex venues like saunas and public spaces, but must also offer MSM strategies to understand risks associated with established partners and to increase condom use in their own homes.

Finally, knowledge of partner HIV serostatus and condom conversations were highly associated in our study, underlining how effective communication skills may underpin multiple risk reduction techniques. Recent studies with MSM in Peru have revealed unacceptably low levels of HIV status communication with sexual partners [41, 42]. While there are no studies explicitly addressing rates of condom negotiation among Peruvian MSM, these conversations were reported in less than 15% of the 1341 sexual encounters in our study. Notably, serostatus conversations were associated with CAI in public sex venues. However, these conversations may have occurred as part of a process of serosorting, suggesting that these encounters were considered lower risk than those involving CAI after no discussion of serostatus. This finding contradicts a study in Portugal that found CAI with a partner whose HIV status was unknown was associated with cruising venues, and may represent a unique target for developing context-specific prevention interventions for MSM in Peru [10]. If informed by recent testing behavior, discussions of HIV serostatus prior to intercourse, even when followed by condomless intercourse, may actually reduce HIV transmission among MSM and illustrate a critical area for improvement in HIV prevention counseling for MSM in Peru [26].

Several limitations to our findings should be considered. First, our results may not be generalizable to all MSM in Lima because we collected a convenience sample of people who volunteered for a trial of rectal STI screening and combination HIV prevention. As the recruitment site is a center for community-based HIV research, our sample is likely to be higher risk, and to report a higher frequency of CAI, than the general MSM population in Peru. Second, because recent condomless receptive anal intercourse was an inclusion criterion for the trial, the sexual orientation and role of participants in our sample were primarily homosexual and *pasivo* or *moderno*. While this characteristic limits the generalizability of our results to MSM who may identify their sexual role as *activo* (insertive) and/or their sexual orientation as hetero- or bisexual, we were able to highlight some differences in site of sexual contact and sexual orientation to be explored in future studies. Finally, our analysis addresses only where participants had sex, and not where they met their sexual partners. As meeting place is likely to have had a significant effect on characteristics like alcohol use (e.g., if a partner was met in a bar or club), this factor may have influenced our findings on issues like substance use.

## Conclusions

Our study shows that CAI and other factors associated with HIV and STI transmission risk, like partner type, substance use, and disclosure of HIV serostatus, vary according to the location where sex occurs. Our findings highlight the importance of addressing how the constellation of these different factors shape the behavioral, biological, social, and environmental contexts of HIV/STI risk and begin to explore how they can be addressed in combined prevention interventions. In the context of Peru’s stable, MSM- and TW-concentrated HIV epidemic that has been stubbornly unresponsive to traditional outreach efforts, novel combination HIV, STI, and substance use prevention interventions must consider how specific environments for sexual contacts between MSM differentially effect condom use and other sexual risk behaviors.

## Data Availability

The datasets generated and/or analyzed during the current study are not publicly available due to restrictions on the publication of human subjects data without participant consent. Study data are available from the corresponding author on reasonable request and pending approval by the UCLA IRB.

## Abbreviations

aPR: Adjusted prevalence ratio
AUD: Alcohol use disorder
CAI: Condomless anal intercourse
CASI: Computer-assisted self-interview
cRAI: Condomless receptive anal intercourse
CT: Chlamydia
EPT: Expedited partner therapy
GC: Gonorrhea
HIV: Human immunodeficiency virus
HSV-2: Herpes Simplex Virus-2
MSM: Men who have sex with men
PR: Prevalence ratio
RPR: Rapid plasma reagin
STI: Sexually transmitted infection
TW: Transwomen

## Footnotes

1 In Lima, “Internet cabins” are cubicle-like, time-based rental spaces offering a computer and Internet access in a semi-private space. As these sites are often used as semi-private commercial sex venues for both spontaneous and coordinated hookups (e.g., through applications like Grindr), we combined Internet cabin encounters with saunas.

## References

[1] UNAIDS. 2016 Peru country factsheet. UNAIDSorg. 2016. Retrieved from http://www.unaids.org/en/regionscountries/countries/peru.

[2] Vargas V. The New HIV/AIDS Program in Peru: The Role of Prioritizing and Budgeting for Results. Washington, DC: World Bank; 2014. Available at http://documents.worldbank.org/curated/en/167721468284339929/pdf/942600WP00PUBL0IV0AIDS0Program0Peru.pdf.

[3] Park H, Konda KA, Roberts CP, Maguiña JL, Leon SR, Clark JL (2016). Risk factors associated with incident syphilis in a cohort of high-risk men in Peru. PLoS One.

[4] Passaro RC, Haley CA, Sanchez H, Vermund SH, Kipp AM (2016). High HIV prevalence and the internet as a source of HIV-related service information at a community-based organization in Peru: a cross-sectional study of men who have sex with men. BMC Public Health.

[5] Rusow JA, Fletcher JB, Reback CJ (2017). Sexual venue choice and sexual risk-taking among substance-using men who have sex with men. AIDS Behav.

[6] van den Boom W, Stolte IG, Roggen A, Sandfort T, Prins M, Davidovich U (2015). Is anyone around me using condoms? Site-specific condom-use norms and their potential impact on condomless sex across various gay venues and websites in the Netherlands. Health Psychol.

[7] Yang C, Latkin C, Tobin K, Seal D, Koblin B, Chander G (2018). An event-level analysis of Condomless anal intercourse with a HIV-discordant or HIV status-unknown partner among black men who have sex with men from a multi-site study. AIDS Behav.

[8] Drumright LN, Weir SS, Frost SDW (2018). The role of venues in structuring HIV, sexually transmitted infections, and risk networks among men who have sex with men. BMC Public Health.

[9] Patel RR, Luke DA, Proctor EK, Powderly WG, Chan PA, Mayer KH (2018). Sex venue-based network analysis to identify HIV prevention dissemination targets for men who have sex with men. LGBT Health.

[10] Gama A, Abecasis A, Pingarilho M, Mendao L, Martins MO, Barros H (2017). Cruising venues as a context for HIV risky behavior among men who have sex with men. Arch Sex Behav.

[11] Al-Ajlouni YA, Park SH, Schneider JA, Goedel WC, Rhodes Hambrick H, Hickson DA (2018). Partner meeting venue typology and sexual risk behaviors among French men who have sex with men. Int J STD AIDS.

[12] Semple SJ, Pitpitan EV, Goodman-Meza D, Strathdee SA, Chavarin CV, Rangel G (2017). Correlates of condomless anal sex among men who have sex with men (MSM) in Tijuana, Mexico: the role of public sex venues. PLoS One.

[13] Godin G, Naccache H, Cote F, Leclerc R, Frechette M, Alary M (2008). Promotion of safe sex: evaluation of a community-level intervention programme in gay bars, saunas and sex shops. Health Educ Res.

[14] Ramanathan S, Deshpande S, Gautam A, Pardeshi DB, Ramakrishnan L, Goswami P (2014). Increase in condom use and decline in prevalence of sexually transmitted infections among high-risk men who have sex with men and transgender persons in Maharashtra, India: Avahan, the India AIDS initiative. BMC Public Health.

[15] Herce ME, Miller WM, Bula A, Edwards JK, Sapalalo P, Lancaster KE (2018). Achieving the first 90 for key populations in sub-Saharan Africa through venue-based outreach: challenges and opportunities for HIV prevention based on PLACE study findings from Malawi and Angola. J Int AIDS Soc.

[16] Allan-Blitz Lao-Tzu, Herrera M. Christina, Calvo Gino M., Vargas Silver K., Caceres Carlos F., Klausner Jeffrey D., Konda Kelika A. (2018). Venue-Based HIV-Testing: An Effective Screening Strategy for High-Risk Populations in Lima, Peru. AIDS and Behavior.

[17] Solomon MM, Mayer KH, Glidden DV, Liu AY, McMahan VM, Guanira JV (2014). Syphilis predicts HIV incidence among men and transgender women who have sex with men in a preexposure prophylaxis trial. Clin Infect Dis.

[18] Perez-Brumer A, Konda K, Salvatierra H (2013). Prevalence of HIV, STIs, and risk behaviors in a cross-sectional community- and clinic-based sample of men who have sex with men (MSM) in Lima. Peru PLoS One.

[19] Passaro Ryan Colby, Segura Eddy R., Perez-Brumer Amaya, Cabeza Jeanne, Montano Silvia M., Lake Jordan E., Sanchez Jorge, Lama Javier R., Clark Jesse L. (2018). Body Parts Matter. Sexually Transmitted Diseases.

[20] Allan-Blitz LT, L SR, Bristow CC, Konda KA, Vargas SK, Flores JA, Brown BJ, Caceres CF, Klausner JD (2017). High prevalence of extra-genital chlamydial or gonococcal infections among men who have sex with men and transgender women in Lima, Peru. Int J STD AIDS.

[21] Lama JR, Lucchetti A, Suarez L, Laguna-Torres VA, Guanira JV, Pun M (2006). Association of herpes simplex virus type 2 infection and syphilis with human immunodeficiency virus infection among men who have sex with men in Peru. J Infect Dis.

[22] Clark JL, Segura ER, Oldenburg CE, Rios J, Montano SM, Perez-Brumer A (2017). Expedited partner therapy (EPT) increases the frequency of partner notification among MSM in Lima, Peru: a pilot randomized controlled trial. BMC Med.

[23] Workowski KA, Berman S (2010). Sexually Transmitted Diseases Treatment Guidelines, 2010. MMWR Recomm Rep.

[24] Delgado JR, Segura ER, Lake JE, Sanchez J, Lama JR, Clark JL (2017). Event-level analysis of alcohol consumption and condom use in partnership contexts among men who have sex with men and transgender women in Lima, Peru. Drug Alcohol Depend.

[25] Ludford KT, Vagenas P, Lama JR, Peinado J, Gonzales P, Leiva R (2013). Screening for drug and alcohol use disorders and their association with HIV-related sexual risk behaviors among men who have sex with men in Peru. PloS one.

[26] Santos-Hovener C, Zimmermann R, Kucherer C, Batzing-Feigenbaum J, Wildner S, Hamouda O (2014). Conversation about Serostatus decreases risk of acquiring HIV: results from a case control study comparing MSM with recent HIV infection and HIV negative controls. BMC Public Health.

[27] Carballo-Dieguez A, Miner M, Dolezal C, Rosser BR, Jacoby S (2006). Sexual negotiation, HIV-status disclosure, and sexual risk behavior among Latino men who use the internet to seek sex with other men. Arch Sex Behav.

[28] Clark J, S J, Segura ER, Salazar X, Konda KA, Perez-Brumer A, Hall E, Klausner JD, Caceres C, Coates TJ (2013). Moderno love: sexual role-based identities and HIV/STI prevention among men who have sex with men in Lima, Peru. AIDS Behav.

[29] Barros AJ, H VN (2003). Alternatives for logistic regression in cross-sectional studies: an empirical comparison of models that directly estimate the prevalence ratio. BMC Med Res Methodol.

[30] Hanley JA, Negassa A, Edwardes MD, Forrester JE (2003). Statistical analysis of correlated data using generalized estimating equations: an orientation. Am J Epidemiol.

[31] Brantley M, Schumacher C, Fields EL, Perin J, Safi AG, Ellen JM (2017). The network structure of sex partner meeting places reported by HIV-infected MSM: opportunities for HIV targeted control. Soc Sci Med.

[32] Logan JJ, Jolly AM, Blanford JI (2016). The Sociospatial network: risk and the role of place in the transmission of infectious diseases. PLoS One.

[33] Vagenas P, Brown SE, Clark JL, Konda KA, Lama JR, Sanchez J (2017). A qualitative assessment of alcohol consumption and sexual risk behaviors among men who have sex with men and transgender women in Peru. Substance use & misuse.

[34] Volkow ND, Baler RD, Compton WM, Weiss SRB (2014). Adverse health effects of marijuana use. N Engl J Med.

[35] MacKillop J, Celio MA, Mastroleo NR, Kahler CW, Operario D, Colby SM (2015). Behavioral economic decision making and alcohol-related sexual risk behavior. AIDS Behav.

[36] Pitpitan EV, Kalichman SC (2016). Reducing HIV risks in the places where people drink: prevention interventions in alcohol venues. AIDS Behav.

[37] Fulcher JA, Shoptaw S, Makgoeng SB, Elliott J, Ibarrondo FJ, Ragsdale A (2018). Brief report: recent methamphetamine use is associated with increased rectal mucosal inflammatory cytokines, regardless of HIV-1 Serostatus. J Acquir Immune Defic Syndr.

[38] Semple SJ, Zians J, Grant I, Patterson TL (2006). Sexual risk behavior of HIV-positive methamphetamine-using men who have sex with men: the role of partner serostatus and partner type. Arch Sex Behav.

[39] Tobin KE, Latkin CA, Curriero FC (2014). An examination of places where African American men who have sex with men (MSM) use drugs/drink alcohol: a focus on social and spatial characteristics. Int J on Drug Pol.

[40] Miller WM, Miller WC, Barrington C, Weir SS, Chen SY, Emch ME (2017). The where and how for reaching transgender women and men who have sex with men with HIV prevention Services in Guatemala. AIDS Behav.

[41] Konda KA, Castillo R, Leon SR, Silva-Santisteban A, Salazar X, Klausner JD (2017). HIV status communication with sex partners and associated factors among high-risk MSM and transgender women in Lima, Peru. AIDS Behav.

[42] Nagaraj S, Segura ER, Peinado J, Konda KA, Segura P, Casapia M (2013). A cross-sectional study of knowledge of sex partner serostatus among high-risk Peruvian men who have sex with men and transgender women: implications for HIV prevention. BMC Public Health.

